# Spectrum of abdominal organ injury in a primary blast type

**DOI:** 10.1186/1749-7922-4-46

**Published:** 2009-12-21

**Authors:** Imtiaz Wani, Fazal Q Parray, Tariq Sheikh, Rauf A Wani, Abid Amin, Imran Gul, Mir Nazir

**Affiliations:** 1 Department of Surgery, S.M.H.S Hospital, Srinagar, Kashmir, India; 2 Department of Surgery SKIMS, Srinagar, Kashmir, India

## Abstract

**Introduction:**

Abdominal organ injury in a primary blast type is always challenging for diagnosis. Air containing abdominal viscera is most vulnerable to effects of primary blast injury. In any patient exposed to a primary blast wave who presents with an acute abdomen, an abdominal organ injury is to be kept in a clinical suspicion.

**Aim:**

Study various abdominal organ injuries occurring in a primary type of blast injury.

Material and methods: All those who had exploratory laparotomy for abdominal organ injuries after a primary blast injury for a period of 10 years from January 1998 - January 2008 were included in this retrospective study.

**Results:**

Total 154 patients had laparotomy for abdominal organ injuries with a primary blast type of injury. Small intestine was damaged in 48 patients (31.1%) followed by spleen in 22.7% cases. 54 patients (35.06%) had more than one organ injured. Liver laceration was present in 30 patients (19.48%). Multiple small gut perforations were present in 37 patients (77.08%). Negative laparotomy was found in 5 patients (3.24%) whereas 3 (1.94%) had re-exploration. Mortality was present in 6 patients (3.89%).

**Conclusions:**

Primary blast injury causes varied abdominal organ injuries. Single or multiple organ damage can be there. Small intestine is commonest viscera injured. Laparotomy gives final diagnosis.

## Introduction

Abdominal organs are always at risk for trauma in primary blast injury (PBI). These are notorious for inflicting multiple organ injury in abdomen. Most common abdominal viscera vulnerable to the PBI are those that containing the air. Proximity to site of blast wave, direction and intensity of primary blast wave (PBW), relative position of body and part of the abdomen struck by primary blast wave and the effect of various contents of abdomen and in the hollow viscera predict type and number of the abdominal organs injured. Clinical findings are varied and may be absent until the onset of complications. Tissue damage from the primary blast wave can be an important cause of occult trauma [1]. PBI may lead to bowel perforation, hemorrhage, mesenteric shear injuries, solid organ lacerations, and testicular rupture. A thorough clinical awareness of presentation of abdominal organ injuries, keen clinical observation complimented with X-ray and sonography abdomens are useful in diagnosis of PBI. These are otherwise always challenging to diagnosis, compounded by potentially conflicting treatment goals [2]. The aim was to study various abdominal organ injuries in a patients who had laparotomy for PBI.

## Materials and methods

This retrospective study was done in S.M.H.S Hospital, Srinagar, Kashmir for a period of 10 years from January 1998 - January 2008. All those patients who had laparotomy for organ injury after PBI were included in this study. Those having laparotomy for other types of blast injury and other than the abdominal organ, injuries had exclusion from the study. Those pateints having associated chest injury or head trauma with abdominal injury were excluded from the study and were referred to SKIMS, Hospital for superspecialisation care.

## Results

During study period, 154 patients had laparotomy for organ injury after having PBI. There were 124 males and 27 females. More than one organ damage was present in 54 patients (35.06%). Maximum time for laparotomy after injury was 11 days in one case who had splenectomy.

58 patients (37.66%) had intestinal perforation and small gut was the commonest organ injured. [Table 1] Small intestine was injured in 48 (31.16%) and large gut in 10 patients (6.49%). Ileum was the most common small gut damaged in 69% (40 patients) followed by a large gut in 10 patients (17.24%), 8 patients (13.79%) having jejunal perforation and rest (5.17%) had duodenal injury. Multiple small gut perforations was present in 37 patients (77.08%), out of which 29 had multiple ileal perforations (78.37%). In large gut, 3 patients (30%) had more than one perforation.

**Table 1:** showing various viscera damaged and surgical procedure done

Small gut perforation	48(31.16%)	Repair in 26 patients
		Colostomy in 2 patients
		Resection anastomosis in 7 patients
		Right hemicolectomy in 2 patients
		Illeostomy in 11 patients
Splenic trauma	35(22.72%)	Splenectomy in 35 patients
(Subcapsular hematoma, laceration and hilar injury)		

Liver laceration	30(19.48%)	Repair in 28 patients
		Gauze packing in 8 patients

Large gut perforation	10 (6.49%)	Colostomy in 3 patients
		Tube caecostomy in 1 patient,
		Repair in 6 patients

Gastric perforation	10(6.49%)	Primary repair in 10 with tube gastrostomy in 4 patients

Kidney damage	10(6.49%)	Nephrectomy in 3 patients patient
(Laceration, hematoma and pedicle avulsion)		Nephorostomy in 1
		Repair in 2 patients

Duodenal trauma	3(1.94%)	Tube duodenostomy in 2 patients
(Laceration and the hematoma)		

Gallbladder trauma	3(1.94%)	Cholecystostomy in 1 patient
		Partial Cholecystectomy in 1 patient
		Cholecystectomy in 1 pateint

Bladder laceration	2(1.29%)	Repair with suprapubic cystostomy in all

Mesenteric laceration	10(6.49%)	Repair in 7 patients
		Resection anastomosis in 3 patients

Retroperitoneal hematoma	10(6.49%)	Midline in 1 patient
		Lateral wall hematoma in 1 patient
		Associated with other visceral trauma in 8 patients

Caecal hematoma with transection of appendix	2(1.29%)	Tube caecostomy with appendectomy in 2 patients

Omental hematoma	1(0.64%)	Omentectomy

Negative laparotomy	5(3.24%)	

Reexploration	3(1.94%)	Posterior diaphragmatic wall bleed after splenectomy-1,
		Missed ileal perforation -1,
		Post operative bleeding from liver laceration -1

In large gut, transverse colon perforation was seen in six patients (60%) and four had caecal perforation (40%). Seven patients (70%) had single perforation. Two patients (1.29%) had transaction of an appendix with a caecal hematoma; site of transaction was near the base of an appendix.

Individual small gut perforation was present in 39 patients(25.32%).4 patients (2.59%) had ileal as well as liver perforation , the 2 patients (1.29%) had ileal perforation and splenic laceration, the 2 patients (1.29%) had associated mesenteric tear, whereas the 1 patient had (0.64%) had an associated gastric , duodenal and pancreatic injury.

Individual large gut perforation was present in six patients (3.89%). Associated with the urinary bladder trauma and the liver laceration was present in 1 patient each (0.64%) whereas 2 patients (1.29%) had associated splenic trauma.

Individual liver laceration was seen in 17 patients (11.03%), the associated gastric perforation, gallbladder injury and large bowel perforation was present in one patient (0.64%) each. Liver laceration associated with the splenic trauma and the kidney trauma was present in two patients each (1.29%).4 patients (2.59%) had associated ileal perforation. Liver laceration with gastric tear and ileal perforation, and the liver tear with gallbladder trauma and duodenal trauma were present in one patient (0.64%) each respectively.

Isolated splenic trauma occurred in 25 patients (16.23%). Splenic laceration with a mesenteric tear, the splenic laceration with a large gut injury, the splenic sub capsular hematoma with a small gut injury, the splenic trauma and a kidney laceration, and the splenic as well as liver laceration was seen in 2 patients each (1.29%).

Retroperitoneal hematoma was seen in 10 patients (6.49%).1 patient (0.64%) had an isolated whereas eight (5.19%) had with associated abdominal visceral damage. Lateral wall retroperitoneal hematoma was present in one patient (0.64%). No retroperitoneal hematoma had exploration in our series. Renal hematoma was present in four patients (2.59%) one patient (0.64%) had associated liver laceration and one patient (0.64%) had with splenic trauma.

Mortality was present in six patients (3.89%). Wound infection was seen in 33 patients (21.42%). two patients (1.29%) had fecal fistula, 1(0.64%) had burst abdomen.3 patients (1.94%) had incisional hernia. 4 patients (4.29%) had adhesion obstruction which were managed conservatively.

## Discussion

PBI produces a spectrum of injury from minor, single to multiple organ injury. Actual incidence of abdominal blast injury is unknown. Explosion-related injuries are infrequently seen in civilian practice [3]. The unique physiologic and medical consequences of blast injuries are often unrecognized and frequently poorly understood [4]. Gas-containing sections of the gastrointestinal tract are most vulnerable to primary blast effect but can also damage solid organs.

In PBI, number and type of the abdominal organs injured are predicted by the proximity to a site of blast, position and posture of a patient, direction of blast wave and whether patient is static or at rest; and number of intervening media in between wave and victim. Age, morphology of abdominal organs, contents in gut may alter PBW direction inside which predict the number and type of viscera damaged and an intensity of injury. Rupture, infarction, ischemia and hemorrhage of solid organs such as the liver, spleen, and kidney are generally associated with very high intensity PBW and proximity of the patient to the origin of PBW. Proximity to origin of primary blast wave is strong predictor of type and number of organ injured.

Clinical presentation of abdominal blast injury may be overt, or subtle and variable. Early signs of gastrointestinal injury include decreased bowel sounds, abdominal tenderness, and rectal bleeding. Abdominal PBI should be suspected in anyone exposed to an explosion with abdominal pain, nausea, vomiting, rebound tenderness, guarding, hematemesis, rectal pain, tenesmus, testicular pain, unexplained hypovolemia, or any findings suggestive of an acute abdomen.

PBI to gut occurs by being proximal to origin and high amplitude of primary blast wave. Gut injury vary in severity from minor sub mucosal hemorrhage, the small perforation to full thickness disruption. Rupture of the bowel may occur as an immediate result of a PBW or this might be a delayed rupture. In small intestine, ileum is usually injured. Number of lacerations can be variable from a single to multiple. Size of laceration varies from, < 1 cm to complete disruption. Each perforation shows ragged margins with surrounding bruising. Laceration is present on the mesenteric side or antimesentric side of gut. Sometimes, disruption of gut is associated with mesenteric tear in continuity.

Large gut laceration is usually present in a transverse colon followed by the caecum. Unlike small gut, single laceration is usually present in a large gut. Caecal injury can be associated with trauma to the vermiform appendix. This can be in the form of transaction of appendix or hematoma of mesoappendix. Transaction of appendix is present near the base. Mesoappendix hematoma can be precipitating event for appendicitis. It should be stressed that if there is any evidence of gut injury, whole gut as well as the mesentery should be thoroughly checked to rule out any additional tears to gut, as these are notorious for causing multiple gut injuries. Sometimes these primary non-perforating intestinal blast injuries evolve into secondary intestinal perforation and can occur up to 14 days following initial blast because of ischemia [5,6].

In PBI, gastric laceration is commonly seen on an anterior wall. These can be often seen associated transverse colon damage being in proximity to stomach. Duodenal trauma is least suspected and difficult to diagnose. A high index of suspicion is always to be kept in a mind. There can be simple laceration of duodenum or can be simply a duodenal hematoma.

Liver trauma in primary blast wave involves sub capsular hematoma or the laceration that can be isolated or associated with other organ injury. Liver laceration can be single, multiple or completely shattered. Laceration can be present on any surface of liver depending mainly on its surface struck by primary blast wave. Organ Injury grade seen in liver was grade II in seven patients, grade III -IV seen in 19 patients, grade V seen in 3 patients and grade VI in 2 patients. Gallbladder damage may occur singly or can be associated with surrounding visceral damage. As per preoperative findings, patient can have a partial cholecystectomy, tube cholecystostomy or rarely cholecystectomy depending on a part of gallbladder damaged.

In splenic trauma, often-primary blast wave inflicts large partial to full thickness laceration or the hilar injury, which deems splenectomy desirable in most of cases. Sub capsular hematoma and small laceration can be present in a small number of cases. Organ injury damage in spleen was grade 1 in 2 patients, grade II in 5 patients, grade III -grade IV seen in 14 patients whereas 9 patients had grade V injury. Renal injury can present as hematoma or laceration, sometimes pedicle avulsion can be there. All those who had nephrectomy had grade IV to grade V laceration

Isolated involvement of omentum in primary blast wave presents as a massive omental hematoma and often requires omentectomy. Retroperitoneal hematoma occurs in isolated manner or may be associated with other visceral injury. These are often bilateral. Sometimes a lateral wall retroperitoneal hematoma is present in a primary blast injury.

Enlarged pathological spleen is prone for easy damage in a primary blast injury. A resistant bleed from posterior diaphragmatic wall can occur after splenectomy, as these have firm adhesions with posterior diaphragmatic wall, accounts for re-exploration which if not diagnosed on table as seen in one case in our series. A thorough check of gut is necessary; a missed gut injury may lead to peritonitis and may account for re exploration seen in one case of our series. A wrong clinical judgment in inexperienced hands being indecisive in repair of liver laceration on table may sometimes turn catastrophe and may bleed profusely postoperatively and deems re-exploration, was present in our one case.

Rapid diagnosis is essential to detect the presence of intra-abdominal injuries across this entire spectrum, as there is substantial morbidity and mortality if treatment is delayed. Sometimes, after PBI with an immediate unexplained clinical instability may lead to laparotomy in haste, which may be negative without any evidence of any visceral injury. Mortality and morbidity determining factors are proximity to site of primary blast, number of viscera damaged, severity of organ damage, age, and time of exploration after occurrence of trauma and the diagnosis and experience of surgeon who performs laparotomy. Three patients with shattered liver having gauze pack had uncontrollable bleeding in postoperative period, the one elderly with systemic co morbidity with multi visceral damage with expanding retroperitoneal hematoma and the two patients with concomitant liver, splenic and retroperitoneal hematoma had death. Intestinal barotrauma is considered as a major source of delayed mortality [7].

Injuries to intra-abdominal organs are to be excluded in all victims of a primary blast wave. A high index of suspicion is required to suspect intestinal barotrauma in PBI. An observational period is useful in exposed patient who show no evidence of injury at the time of admission but may manifest later on. Physical examination remains the initial step in diagnosis but has limited utility under select circumstances and findings may not be reliable always. Early radiographs of the abdomen may reveal free air under the diaphragm or air in the lumen of the intestine and indicate significant abdominal injury and are highly beneficial [8]. Sometimes the emergence of these radiological signs is delayed for several days.

Definite preoperative diagnosis and the decision to have surgical intervention are based on keen clinical assessment and observation and the use of plain radiograph of abdomen and FAST (Focused Assessment with Sonography for Trauma). Conservative treatment in salvageable solid visceral injury in primary blast injury in our setting is restricted as a lack of easy availability of advanced imaging techniques and intensive care unit, sophisticated resuscitation measures and the invasive monitoring facilities. Moreover, multiple organ injury in a number of individual patients in this series did not favored conservative management in our settings. Laparotomy continues to be decisive factor in final diagnosis.

## Conclusion

PBI causes varied abdominal organ injuries. Single or multiple organ damage can be there. Intestines as well as solid viscera are prone for damage. Small intestine is commonest viscera damaged. Multiple perforations are present commonly in a small gut. An awareness of presentation of pattern of injuries occurring in a primary injury can make early diagnosis. Observation period for those who have been very close to the site of blast even without any evident injury is quite important, as it is not only the pallets but also even the blast waves, falling of objects, stampede which can inflict very serious trauma to these patients. Most of the times laparotomy may reveal even the most concealed injuries.

## Competing interests

The authors declare that they have no competing interests.

## Authors' contributions

IW: took acquisition of data, compilation of relevant literature, formatting, revision, drafted the preliminary and final manuscript. FQ: helped in drafting, acquisition and revision of manuscript TS, RW AA, and IG:helped in acquisition of data and revision of manuscript. MN:helped in final drafting and revision of manuscript. All authors have read manuscript and approved the final version of manuscript.
